# The Adoption of "Summer Time."

**Published:** 1920-03-13

**Authors:** 


*To the Editor of* THE HOSPITAL.

### The Adoption of "Summer Time."

*To the Editor of* THE HOSPITAL.

SIR,—I note in a recent issue of THE HOSPITAL a paragraph relating to summer-time, in which it is stated that it is in force in other countries. You omit to state that U.S.A., Canada, Australia, Denmark, and other countries, including, I think, Germany, that tried the scheme promptly dropped it, a short experience proving the many disadvantages outweighed any possible advantage. Apart from nearly all engaged in agriculture (who number millions) who strongly denounce the measure, it has been adversely criticised by many medical men and numbers of school-teachers for its most pernicious effect on children of school age. It also inflicts the greatest hardship on working-class wives, who are compelled by the Act to rise an hour earlier, but are seldom able to get to bed any sooner than usual, thus lengthening their already long day. Why cannot the people of this country be left alone? If those who like early rising are at liberty to do so, why should they be allowed to inflict their fad on others. With the short hours now worked it cannot be said that anyone (except housewives) have not sufficient leisure. There is another point. The alteration of the clock makes the most crowded times of travelling come in the hot hours of the afternoon (five being really four), and those who have experienced travelling seventeen and upwards in a compartment at four in the afternoon know to their cost one of the blessings (?) of summer-time. I enclose my card and remain, Yours faithfully,

LIBERTY.

